# Efficacy of vinblastine in central nervous system Langerhans cell histiocytosis: a nationwide retrospective study

**DOI:** 10.1186/1750-1172-6-83

**Published:** 2011-12-12

**Authors:** Sophie Ng Wing Tin, Nadine Martin-Duverneuil, Ahmed Idbaih, Catherine Garel, Maria Ribeiro, Judith Landman Parker, Anne-Sophie Defachelles, Anne Lambilliotte, Mohamed Barkaoui, Martine Munzer, Martine Gardembas, Jean Sibilia, Patrick Lutz, Renato Fior, Michel Polak, Alain Robert, Olivier Aumaitre, Dominique Plantaz, Corinne Armari-Alla, Thierry Genereau, Perrine Marec Berard, Ghislain Nokam Talom, Jean-Loup Pennaforte, Hubert Ducou Le Pointe, Marie-Anne Barthez, Gérard Couillault, Julien Haroche, Karima Mokhtari, Jean Donadieu, Khê Hoang-Xuan

**Affiliations:** 1 APHP-UPMC, Service de neurologie 2-Mazarin, Groupe Hospitalier Pitié-Salpêtrière, Paris, France; 2 Service de neuroradiologie, Groupe Hospitalier Pitié-Salpêtrière, Paris, France; 3 Service de radiologie, Hôpital Trousseau, Paris, France; 4 Commissariat à l'énergie atomique, Orsay, France; 5 Service hémato-Oncologie pédiatrique, Hôpital Trousseau, Paris, France; 6 Unité d'oncologie pédiatrique, centre Oscar-Lambret, Lille, France; 7 Service d'hématologie pédiatrique, CHU de Lille, Lille, France; 8 Centre de référence des histiocytoses, Registre des histiocytoses, Service d'hémato oncologie pédiatrique, Hôpital Trousseau, Paris, France; 9 Service Hémato - Oncologie Pédiatrique CHU Reims, France; 10 Service Hémato - Oncologie Pédiatrique CHU Angers, France; 11 Service de rhumatologie, Centre national de références des maladies auto-immunes systémiques, hôpital de Hautepierre, CHU de Strasbourg, France; 12 Service de pédiatrie, CHU de Strasbourg, France; 13 Service de médecine interne, hôpital Béclère, Clamart, France; 14 Service d'endocrinologie pédiatrique, Hôpital Necker APHP, France; 15 Service d'hémato oncologie Pédiatrique CHU Purpan Toulouse, France; 16 Service de médecine interne, CHU de Clermont-Ferrand, France; 17 Unité d'hémato Oncologie Pédiatrique CHU de Grenoble, France; 18 Médecine Interne, Nouvelle Clinique Nantaise, Nantes, France; 19 Institut d'hémato Oncologie Pédiatrique CHU de Lyon, France; 20 Polyclinique de Deauville, 14113 Cricqueboeuf, France; 21 Service de médecine interne, CHU de Reims, France; 22 Neurologie pédiatrique, hôpital Clocheville, CHRU Tours, France; 23 Service de pédiatrie, CHRU de Dijon, France; 24 Service de médecine interne, Groupe Hospitalier Pitié-Salpêtrière, Paris, France; 25 Service de neuropathologie, Groupe Hospitalier Pitié-Salpêtrière, Paris, France

## Abstract

**Background:**

Vinblastine (VBL) is the standard treatment for systemic Langerhans cell histiocytosis (LCH), but little is known about its efficacy in central nervous system (CNS) mass lesions.

**Methods:**

A retrospective chart review was conducted. Twenty patients from the French LCH Study Group register met the inclusion criteria. In brief, they had CNS mass lesions, had been treated with VBL, and were evaluable for radiologic response.

**Results:**

The median age at diagnosis of LCH was 11.5 years (range: 1-50). Intravenous VBL 6 mg/m^2 ^was given in a 6-week induction treatment, followed by a maintenance treatment. The median total duration was 12 months (range: 3-30). Eleven patients received steroids concomitantly. Fifteen patients achieved an objective response; five had a complete response (CR: 25%), ten had a partial response (PR: 50%), four had stable disease (SD: 20%) and one patient progressed (PD: 5%). Of interest, four out of the six patients who received VBL without concomitant steroids achieved an objective response. With a median follow-up of 6.8 years, the 5-year event-free and overall survival was 61% and 84%, respectively. VBL was well-tolerated and there were no patient withdrawals due to adverse events.

**Conclusion:**

VBL, with or without steroids, could potentially be a useful therapeutic option in LCH with CNS mass lesions, especially for those with inoperable lesions or multiple lesions. Prospective clinical trials are warranted for the evaluation of VBL in this indication.

## Background

Langerhans cell histiocytosis (LCH) is a rare disease characterized by an accumulation of Langerhans cells [1]. Clinical presentation ranges from isolated benign localization to multisystemic aggressive lesions. Although it is most common in children under 15 years of age, it may occur at any age [2-4]. Central nervous system (CNS) complications of LCH occur in 1-11% of patients [4-6] and can be subdivided clinically into two subtypes, each corresponding with a distinct pathophysiology. The first type is the 'neurodegenerative-like' form, characterized by neuronal cell loss and a progressive cerebellar ataxia, frequently combined with pyramidal syndrome and cognitive dysfunction [7-12]. The second type is the tumoral or mass lesions-type presenting as unique or multiple contrast-enhancing space-occupying lesions [13-16]. In this latter form, the lesions can be located anywhere in the CNS but are most commonly found in the hypothalamo-pituitary region. Optimal treatment for LCH with CNS mass lesions is not yet well defined, and depends on the site of the disease [5]. Vinblastine (VBL) chemotherapy is the standard treatment for aggressive systemic LCH [17], but little is known about its efficacy in LCH with CNS mass lesions. In order to evaluate the efficacy and safety of VBL, we retrospectively studied patients from the French LCH register who had received VBL for LCH with CNS mass lesions.

## Methods

Patients with CNS mass lesions were identified from the French LCH Study Group register. This database was initially created for a retrospective study of patients with LCH between 1983 and 1993, but from 1994 enrolment into the database was conducted prospectively. Since 2008 this collection of data has been recognized as a national register by the French health authorities and has been verified against multiple separate sources [18]. The database has been declared to the French computer watchdog authorities (Comité Consultatif pour le Traitement de l'Information en matière de Recherche pour la Santé [CCTIRS] and Commission Nationale Informatique et Liberté [CNIL]), and the patient must provide informed consent to be included in the register. Data monitoring, based on medical charts, was conducted by a clinical research associate who visited each center. Patients listed in the register were included in the study if they had: i) LCH with CNS mass lesions defined as the presence of a contrast-enhancing space-occupying CNS lesion occurring in a patient with either proven systemic LCH or who meets the pathologic criteria for LCH on CNS biopsy according to the histiocyte society (CD1a positive cells or Birbeck granules)[19]; ii) received treatment with VBL either as initial or salvage treatment; and iii) had measurable disease with lesions visible after contrast-enhanced magnetic resonance imaging (MRI) and were evaluable for tumor response (i.e. availability of pre and post-therapeutic MRIs for review). Imaging studies performed before and after treatment with VBL were reviewed independently by three investigators (NMD, SNWT and CG).

The primary endpoint was the radiologic response to VBL therapy. Response was assessed by determining the product of the two largest perpendicular diameters of the lesion on the axial T1 planes of the MRI scan, as previously reported by Macdonald [20]. A complete response (CR) was defined as complete disappearance of all mass lesions; a partial response (PR) was defined as a greater than 50% reduction in the size of all measurable mass lesions, and patients must be on stable or reduced doses of corticosteroids and show a stable or improved neurologic status. Progressive disease (PD) was defined as a greater than 25% increase in the size of measurable mass lesion(s) or as tumor-related neurologic deterioration. Stable disease (SD) was defined as any other clinical status not meeting the criteria for CR, PR, and PD. Secondary endpoints included event-free survival, number of relapses, endocrine sequelae, death and overall survival. Event-free survival was defined as the time from start of VBL treatment until the event (death, relapse or progression), or the date of last examination if no event occurred. Initial failure (PD) was considered as an event and, in this instance, the time-to-event was equal to zero. Relapse was defined as disease progression after an initial favorable response. The Kaplan-Meier method was used to estimate overall survival and event-free survival. The cut-off date for data analysis was November 15, 2010.

## Results

### Patients

Among 1411 patients from the French LCH Study Group register, 57 were identified with CNS Langerhans cell histiocytic mass lesions. Of these, 37 were excluded: two had been treated only with surgery, four had received 2-chlorodeoxyadenosine (2-cda) as initial therapy, four were under observation, ("watchful waiting"), two had received other chemotherapy and radiotherapy treatments, and the remaining 26 did not have MRI scans accessible for review. The retained 20 patients fulfilled the inclusion criteria of the study, including having received VBL treatment and were evaluable for an objective response. CNS lesions were documented by MRI imaging in all patients, and additionally in 9 patients by CNS mass lesion biopsy to confirm tumor immunohistochemistry.

### Patient characteristics

The main clinical characteristics of the patients are summarized in Table 1. Of the 20 patients, 12 were female and 8 male. The median age of LCH diagnosis onset was 11.5 years (range: 1-50) and the median age of neurologic involvement diagnosis was 12 years (range: 3-53). Neurologic diagnosis were simultaneous with the first occurrence of LCH in 12 patients and occurred during a reactivation in 8 patients (after a median delay of 3.7 years since the first occurrence of LCH).

**Table 1:** Patient demographic and clinical characteristics

Patient	Sex	Age at diagnosis of LCH (years)	Age at diagnosis of CNS involvement (years)	Localisation of CNS mass lesion	Other sites of LCH involvement	Symptoms
1	F	10	12	hp	Bone, skin	Diabetes insipidus

2	F	50	53	p (brainstem)	mastoid	Cerebellar ataxia

3	F	26	28	hp	None	Cognitive impairment, bulimia, diabetes insipidus

4	M	9	9	hp	None	Diabetes insipidus

5	M	21	34	p (multifocal: frontal and brainstem)	Bone, lung	Cerebellar ataxia, cranial nerve palsies, hemiparesis, diabetes insipidus

6	F	2	3	hp	Skin	Diabetes insipidus

7	M	41	44	hp	Lung	Cognitive impairment, diabetes insipidus

8	F	3	3	hp	Skin, bone, thyroid	Diabetes insipidus

9	F	7	7	hp	Skin, lung	Diabetes insipidus

10	F	1	10	hp	Skin, bone,	Visual field defect

11	F	4	4	hp and m	Bone	Diabetes insipidus

12	M	26	30	hp and p (brainstem)	Skin, lung, parotid, liver	Hemiparesis, headaches

13	M	35	35	hp	Bone	Diabetes insipidus

14	F	15	16	hp	None	Headaches

15	F	2	12	p (multifocal: temporal, frontal, parietal lobes)	Bone, skin	Diabetes insipidus

16	M	28	28	hp	Bone	Cognitive impairment, diabetes insipidus

17	F	15	15	hp	None	Visual field defect, diabetes insipidus

18	M	1	5, 5	hp	Bone, skin	Diabetes insipidus

19	F	12	12, 1	hp	None	Diabetes insipidus

20	M	11	11	p (cerebellum)	None	Cerebellar ataxia

CNS = central nervous system, F = female, hp = hypothalamo-pituitary region involvement, LCH = Langerhans cell histiocytosis, M = male, p = parenchymal involvement, m = meningeal involvement.

The CNS mass lesions were solitary (n = 15) or multifocal (n = 5). Lesions were located solely in the hypothalamo-pituitary region in 14 patients and were solely extrahypothalamic in 4 patients; 2 patients had both hypothalamic and extrahypothalamic lesions. All three patients with brainstem mass lesions were adults. In addition to a mass lesion, three patients had typical bilateral non-enhancing lesions of the cerebellum white matter, which are usually observed in the neurodegenerative form of LCH (patients #2, #5 and #6). Symptoms of CNS disease were linked to the site of the CNS lesions (diabetes insipidus, cognitive impairment, seizures, hemiparesis, cerebellar ataxia, cranial nerve palsies). CNS disease was associated with multisystemic LCH in 13 patients (bone, skin and lung involvement) and isolated in 7 patients. Eighteen patients were previously untreated before VBL therapy, and two patients were previously treated (patient #10 with steroids and patient #5 with combination chemotherapy of methotrexate plus etoposide). Intravenous VBL was delivered at a standard dose (6 mg/m^2^), given once weekly for 6 weeks (induction treatment) followed by a maintenance dose every 3 weeks. All patients received the 6-week induction treatment; the median duration of maintenance treatment was 12 months (range 3-30). Eleven patients were already receiving steroids prior to initiation of VBL or received steroids concomitantly to VBL as part of the chemotherapy regimen (Table 2).

**Table 2:** Response to treatment and outcome

Patient	Treatment duration (months)^a^	Concomitant steroids (Y/N)	Radiologic response	Change to therapeutic regimen after radiologic evaluation (Y/N) [details]	Relapse (Y/N) [details]	Treatment for relapse [details]	Endocrine sequelae	NDS/cognitive impairment (time since CNS mass lesions)	Final MRI features	Vital status at follow-up visit [duration of follow-up (years)]^d^
1	21	Y	CR	N	N		DI, GHD	None	Normal	L [9.0]

2	3	N	PD	Y [therapy withdrawn]	N		DI	NDS (simultaneously)	Neurodegenerative lesions	D [4.4]

3	17	N	CR	N	N		Panhypopituitarism	NDS (about 1 y after mass lesion)	Atrophy	L [6.8]

4	12	N	PR	N	N		DI, GHD, obesity, hypothalamic syndrome	Behavioral disturbance - several months after the end of therapy	Residual lesions^c^	L [6.7]

5	13	Y	SD	N	Y [brain stem, month 12]	IC plus autograft [resulting in PR, subsequent death from sepsis]	DI	NDS (simultaneously)	Tumoral lesion and neurodegenerative lesion	D [7.1]

6	13	N	PR	N	N		DI	NDS (3 years after mass lesion)	No tumoral lesion. Neurodegenerative lesions	L [7.2]

7	18	Y	SD	N	Y [temporal lobe, month 16]	VBL [death 1 month after VBL]	DI	NDS (simultaneously)	Tumoral lesion and neurodegenerative lesion	D [4.4]

8	30	Y	PR	N	Y [hypothalamus, month 15]	VBL	DI	None	Residual lesions^c^	L [10.9]

9	12	N	SD	N	N		DI	None	Residual lesions^c^	L [3.8]

10	18	Y	CR	N	N		DI, morbid obesity, hypothalamic syndrome	None	Residual lesions^c^	L [7.4]

11	7	Y	PR	N	Y [hypothalamus, month 19]	VBL [PR)	DI	None	Residual lesions^c^	L [1.4]

12	6	Y	SD	N	Y [brain stem, month 47]	2-cda [relapse at 5 months] Subsequent treatment with autograft [CR/remission]	DI	None	Residual lesions^c^	L [8.3]

13	10	Y	PR	N			DI	None	Normal	L [10.0]

14	12	N	PR	N	Y [hypothalamus month 3]	VBL, [VBL allergy^b^]Then RT [failure], then 2-cda [CR]	Panhypopituitarism	None	Residual lesions^c^	L [10.9]

15	12	Y	PR	N			DI, GHD	None	Normal	L [21.3]

16	5	Y	CR	N	Y [brainstem, month 20]	2-cda [frank progression leading to death]	DI	Cognitive impairment - progressive after end of initial therapy	Disease progression (to brain stem)	D [1.9]

17	12	Y	PR	N	Y [bone only)	VBL plus steroid	DI	None	Residual lesions^c^	L [7.3]

18	4 (ongoing)	Y	PR	N	N		DI	None	Residual lesions^c^	L [0.4]

19	12	Y	PR	N	N		Panhypopituitarism	None	Residual lesions^c^	L [5.0]

20	11	Y	CR	N	N		None	None	Normal	L [6.9]

2-cda = 2-chlorodeoxyadenosine, CR = complete response, D = deceased; DI = central diabetes insipidus, IC = intensive chemotherapy, GHD = growth hormone deficiency, L = living; MRI = magnetic resonance imaging; N = no, NDS = neurodegenerative syndrome; PD = progressive disease, PR = partial response, RT = radiotherapy, SD = stable disease, VBL = vinblastine, Y = yes.

^a ^The duration of treatment is calculated from the start of VBL to the end of the maintenance treatment, i.e. the last pulse of VBL and/or the last steroid dose.

^b ^Repeated skin risk after VBL pulses, increasing after repeated injections.

^c ^Residual lesion are considered if the mass lesion remained unchanged during sequential MRI at least during a 6-month interval.

^d ^Duration is calculated from the time of diagnosis of CNS mass lesions.

### Primary endpoint: response to treatment

**Figure 1:**
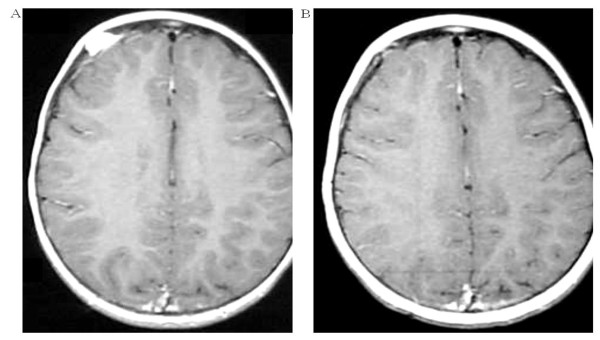
**Complete response to vinblastine chemotherapy**. Dura mater lesion. Axial T1-weighted magnetic resonance imaging (MRI) with gadolinium before treatment with vinblastine (A) and 8 months after treatment initiation showing a complete response (B).

**Figure 2:**
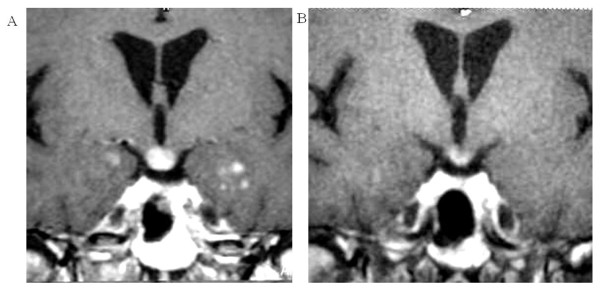
**Partial response to vinblastine chemotherapy**. Hypothalamic and temporal lesions. Coronal T1-weighted magnetic resonance imaging (MRI) with gadolinium before treatment with vinblastine (A) and 12 months after treatment initiation, showing a partial response (B).

**Figure 3:**
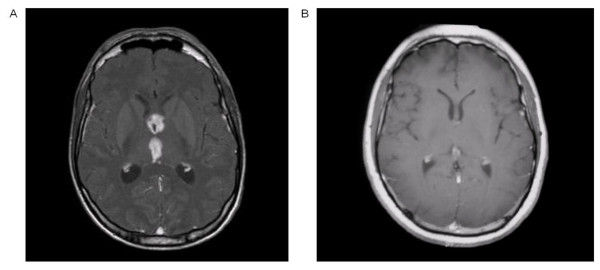
**Complete response to vinblastine chemotherapy**. Third ventricle lesion. Axial T1-weighted magnetic resonance imaging (MRI) with gadolinium before vinblastine treatment (A) and 6 months after treatment initiation, showing a complete response (B).

Fifteen patients achieved an objective response, including five complete responses (CR 25%) and 10 partial responses (PR 50%); four patients had stable disease (SD 20%) and one had progressive disease (PD 5%). Of interest, four out of the six patients who received VBL but not steroids achieved an objective response (1 CR, 3 PR, 1 SD, 1 PD; Figures 1, 2 and 3). After evaluation of the initial response, the 10 patients with a partial response continued VBL maintenance therapy until the end of the therapy or in one case, until disease progression (Table 2).

### Secondary endpoints

Treatment with VBL was well-tolerated. The most severe adverse events were mild peripheral neuropathy (grade 2; n = 2, patients #10 and #12) and increased liver enzymes (grade 2; n = 2, patients #14 and #16). There were no reports of patients withdrawing from treatment as a result of adverse events.

**Figure 4:**
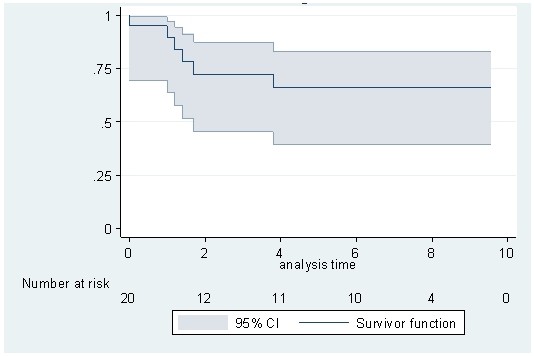
**Event-free survival calculated by the Kaplan-Meier method**. Events are progressive disease or any new progression after initial control of the CNS lesion. Duration is expressed in years.

After a median follow-up of 6.8 years (from VBL-treatment onset), the 2- and 5-year event-free survivals were 67% and 61%, respectively, with a plateau after 3 years (Figure 4). At the time of analysis, seven patients had relapsed. Details of therapy for relapse are included in Table 2: three patients were retreated with VBL chemotherapy and responded, one patient underwent radiotherapy but with no improvement, but did respond thereafter to treatment with 2-cda. Two patients responded to an intensive chemotherapy regimen with hematopoietic stem cell support (the latter after failure of 2-cda treatment) but one among the two died from complications of neurodegenerative CNS LCH. Finally one patient did not respond to 2-cda treatment and experienced rapid tumoral progression leading to death.

The 5-year overall survival rate was 84% (95% CI: 58-94%). Four patients died, at 1, 14, 21 and 24 months after the initiation of VBL treatment. Of note, three of these patients (2# 5# 16#) had a mass lesion in the brainstem and two (patient 2# and 5#) had bilateral non-enhancing lesions of the cerebellum white matter. The immediate cause of death was a pulmonary embolism (n = 1) (patient 7#), sepsis (n = 1) (patient 5#) and neurologic disease progression (n = 2) (patient 5# and 16#). Endocrine sequelae were observed in 19 patients: with central diabetes insipidus (CDI) in 19 patients, growth hormone deficiency in 6 patients and panhypopituitarism in 3 patients. CDI was always present at the diagnosis of CNS mass lesions and persisted, even in the case of CR. The age of patients at diagnosis, specifically those over 18 years old, was associated with a poorer outcome compared with those younger than 18 years old, both in terms of event-free survival (5-year event-free survival rates of 28% vs 90%; p = 0.005) and overall survival (5-year overall survival rate of 57% vs 100%; p = 0.0052).

## Discussion

Among 20 patients with LCH and CNS mass lesions, a 75% objective response rate was observed following VBL chemotherapy. Because the majority of patients received steroids at the same time (as an anti-inflammatory or as part of their chemotherapy regimen), it is difficult to attribute the treatment response solely to VBL. However, in the subgroup if patients who did not receive any steroids in their initial treatment, the objective response rate was comparable with the overall response rate of the steroid-treated group (2/3 of the patients i.e. 4 responders out of 6 patients). This suggests that VBL, either as monotherapy or in combination with steroids, is highly active in LCH with CNS mass lesions. Additionally, patients who respond do not appear to become desensitized to VBL over time: three patients who relapsed after VBL treatment were successfully retreated at recurrence with the same VBL regimen. Our results confirm and extend those of case reports; an objective response to VBL as primary or salvage therapy was observed in four out of five patients in LCH with CNS mass lesions [21-25]. Contrary to its effect on mass lesions, VBL seems to have no effect on neurodegenerative lesions and their related symptoms, which seem to arise via pathophysiologic mechanisms distinct from CNS mass lesions [7]. Interestingly, the patients in this study that demonstrated a mixed form of LCH worsened.

VBL, a vinca alkaloid, is a key drug in the treatment of multisystem LCH, where response rates of up to 70% have been achieved [17,26], which is comparable with the response rate observed in our present study for CNS mass lesions. This suggests that VBL penetrates the blood-brain barrier sufficiently so as to have a therapeutic effect on CNS mass lesions. In addition, VBL is well tolerated and can be delivered for a prolonged time without significant cumulative toxicity, as observed in the present study and elsewhere [26].

The purine substrate analogue 2-cda has been also been shown to be similarly effective for systemic LCH [27], but it is widely considered more appropriate as a second-line therapy [28]. Paradoxically, its use has been more frequently reported than the use of VBL in patients with CNS mass lesions: Dhall et al reported a series of 12 patients treated with 2-cda who achieved an objective response (8 CR and 4 PR) [29]. Similarly to the patients in this study, those in the Dhall group had CNS lesions primarily in the hypothalamo-pituitary region (n = 10), while the remaining two patients had extra-hypothalamic lesions located in the dura mater. While 2-cda treatment yielded a high response rate, it was also associated with substantial toxicity, especially prolonged bone marrow suppression previously described in other studies [30]. In light of this, 2-cda would be likely to be best utilized as second-line chemotherapy after VBL in relapsed or treatment-refractory patients. Anecdotal cases of LCH with CNS mass lesions treated with intensive chemotherapy plus hematopoietic stem cell support have been reported, with a prolonged response observed in some patients, but absence of response in others [31].

## Conclusion

Our results suggest that VBL is a therapeutic option for CNS mass lesions in LCH, and support the continued evaluation of VBL in prospective trials. Chemotherapy with VBL appears to be of particular interest for the treatment of lesions in non-operable locations, multifocal lesions or secondary CNS lesions in the setting of systemic active LCH disease.

## Competing interests

The authors declare that they have no competing interests.

## Authors' contributions

SNWT analyzed the data and wrote the manuscript. NMD, AI, CG, MR, JLP, ASD, AL, MB, MM, MG, JS, PL, RF, MP, AR, OA, DP, CA, GC, TG, PMB, GNT, JLP, HDP, MAB, JH, and KM revised the manuscript. JD designed the study, analyzed the data and revised the manuscript. KHX designed the study and revised the manuscript. All authors read and approved the final manuscript.
